# Macro- and microplastics affect cold-water corals growth, feeding and behaviour

**DOI:** 10.1038/s41598-018-33683-6

**Published:** 2018-10-17

**Authors:** L. Chapron, E. Peru, A. Engler, J. F. Ghiglione, A. L. Meistertzheim, A. M. Pruski, A. Purser, G. Vétion, P. E. Galand, F. Lartaud

**Affiliations:** 1 Sorbonne Université, CNRS, Laboratoire d’Ecogéochimie des Environnements Benthiques, LECOB, F-66650 Banyuls-sur-Mer, France; 2 Sorbonne Université, CNRS, Laboratoire d’Océanographie Microbienne, LOMIC, F-66650 Banyuls-sur-Mer, France; 3 The Alfred Wegener Institute, Helmholtz Centre for Polar and Marine Research, Bremenhaven, 27570 Germany

## Abstract

Plastic contamination is now recognized as one of the most serious environmental issues for oceans. Both macro- and microplastic debris are accumulating in surface and deep waters. However, little is known about their impact on deep marine ecosystems and especially on the deep-sea reefs built by emblematic cold-water corals. The aim of this study was to investigate whether plastics affected the growth, feeding and behaviour of the main engineer species, *Lophelia pertusa*. Our experiments showed that both micro- and macroplastics significantly reduced skeletal growth rates. Macroplastics induced an increased polyp activity but decreased prey capture rates. They acted as physical barriers for food supply, likely affecting energy acquisition and allocation. Inversely, microplastics did not impact polyp behaviour or prey capture rates, but calcification was still reduced compared to control and *in situ* conditions. The exact causes are still unclear but they might involve possible physical damages or energy storage alteration. Considering the high local accumulation of macroplastics reported and the widespread distribution of microplastics in the world ocean, our results suggest that plastics may constitute a major threat for reef aggradation by inhibiting coral growth, and thus jeopardise the resilience of cold-water coral reefs and their associated biodiversity.

## Introduction

Among the anthropogenic activities that significantly affect marine ecosystems, the release of plastic wastes is a key challenge for the preservation of biodiversity and associated resources^1^. Plastic debris most often disseminate from land and accumulate in the oceans where they represent 60 to 80 percent of marine litter^2^. Macroplastic pollution was recognized as a major threat decades ago and has been surveyed within the frame of the Marine Strategy Framework Directive^3^. Impacts from exposure to these plastics (e.g. entanglement, ingestion) have been observed on different marine species^4^, including benthic organisms, which can suffer from plastics coverage of tissues, or direct physical abrasion following exposure^5^. Microplastics (i.e., plastic fragments <5 mm) have been a topic of discussion in recent years, and the potential threats they pose to marine ecosystems a focus of active debate^6–8^. These small plastics can originate from primary sources, manufactured already at microscopic size, or be derived from the fragmentation of macroplastics. Microplastics in marine environments mainly consist of polypropylene (24%), low-density polyethylene (21%), polyvinyl chloride (19%) and high-density polyethylene (17%)^9^. The hazards posed to marine fauna by this material are multiple, including the risk of direct ingestion, entanglement, transfer and bioaccumulation within the food web, and as vectors of potentially harmful heavy metal, organic pollutant, and microbial transport^10–12^. At the level of physiological functioning microplastics have been shown to impair feeding in copepods and crabs^13,14^, affect the larval development of oysters^15^, reduce the energy storage of lugworms^16^, accumulation in the gut cavity and possible bleaching and necrosis of shallow-water corals^17,18^. However, these results need to be considered with caution considering that they originate from laboratory studies with artificially high concentrations of plastics that may not be representative of the natural environment^19^. But, as highlighted by Huvet *et al*.^20^, the use of high concentrations in ecotoxicological studies can be viewed as a proof-of-concept to assess the potential risk of emerging pollutants and determine adequate biomarkers.

The great majority of studies on microplastics have focused on shallow-water environments, although the deep-sea is now recognised as a major sink for plastic debris^21–23^. Plastics fragments are routinely colonized by microbial biofilms which influence hydrophobicity and buoyancy, and therefore impact vertical transport velocities^24^. Sinking to the deep-sea is size-dependant and larger microplastics have a greater probability of reaching the sea floor^25^. Plastics particularly aggregate in submarine canyons where they represent the dominant part of the marine litter^26^. In the Mediterranean canyons, plastic debris represent overall ca. 70% of the observed wastes, with macro debris concentrations reaching >1 item 100 m^−1^ ^27,28^. Deep-sea fauna such as gastropods, echinoderms, crabs and cold-water corals have been shown to ingest microplastics^29,30^, and they could thus be impacted by plastic pollution.

Cold-water (also called deep-sea) corals are often found in submarine canyons where they form reefs and structures that provide niches and nursery grounds for a variety of other species, including commercial fish and decapods. Cold-water corals play a key role in deep-sea ecosystems^31,32^ by supporting deep-sea biodiversity. They also support ecological services such as paleo-climate indicators, sources of material for the production of novel pharmaceutical compounds, and as sinks for CO_2_ sequestration^33^.

The structural complexity of coral reefs leads to a preferential accumulation of plastics within cold-water corals habitats^28^, and it has been reported that at least some deep-sea corals species ingest microplastics^29^. However, the effects of plastics on deep sea corals have not been investigated so far. The aim of this study was to test whether plastics affected the growth, feeding and behaviour of the main deep engineer species found in European waters, *Lophelia pertusa* (recently synonymised to *Desmophyllum pertusum*^34^). We conducted aquaria based experiments to measure the effects of both macro- and microplastics on skeletal growth, prey capture rates and polyp activity, and thus address for the first time the question of deep-sea reef sustainability in a plastic world.

## Results

### Capture rates and polyp activity

**Figure 1:**
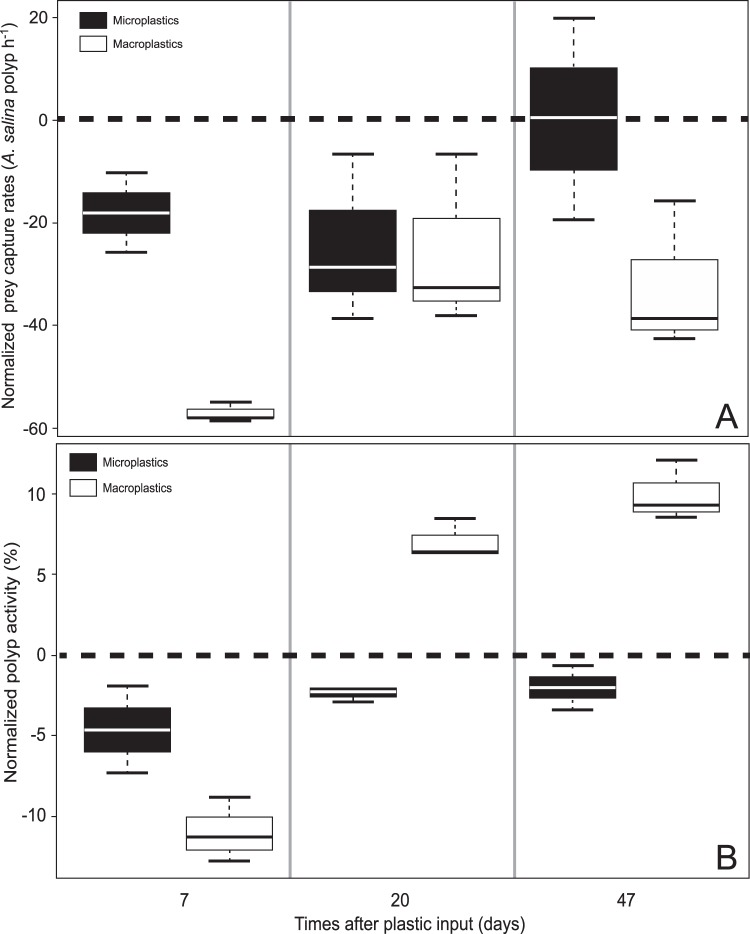
(**A**) Average net capture rates of *A*. *salina* per polyp during the first hour of feeding and (**B**) polyp activity after 7, 20 and 47 days of experiment. Values are normalised against controlled conditions (dotted line). All values presented are the median and quartiles.

Under control condition, polyps had an average capture rate of 72.25 ± 19.36 *A*. *salina* polyp^−1^ during the first hour and 32.17 ± 2.28% of the polyps were active on the camera surveys. To analyse the impact of both macro- and microplastics on capture rates and polyp activity, the experimental data were expressed relative to control conditions (Fig. 1). All statistical values are represented in Table 1.

**Table 1:** Differences in capture rates and polyp activity of the cold-water coral *Lophelia pertusa* between different experimental conditions (control, macroplastic and microplastic exposure).

		Capture rates (*A*. *salina* polyp h^−1^)				Percentage of active polyps (%)			
		Control		Macroplastics		Control		Macroplastics	
	days	Mean ± SD	K-W χ^2^	Mean ± SD	K-W χ^2^	Mean ± SD	K-W χ^2^	Mean ± SD	K-W χ^2^
Microplastics	7	−18 ± 7.72	3.971*	+36.59 ± 6.30	3.971*	−4.64 ± 2.74	2.333	+6.35 ± 0.82	3.971*
	20	−24.43 ± 16.42	3.971*	+1.31 ± 2.58	0.441	−2.48 ± 0.36	4.5*	−9.55 ± 1.57	4.091*
	47	+0.31 ± 19.69	0.048	+32.83 ± 12.07	3.971*	−2.03 ± 1.37	3.857*	−11.99 ± 0.74	3.971*
Macroplastics	7	−57.10 ±1.90	3.857*			−10.99 ± 1.98	3.971*		
	20	−25.74 ± 16.94	3.857*			+7.07 ± 1.21	4.5*		
	47	−32.52 ± 14.53	3.971*			+9.96 ± 1.89	3.971*		

Statistical differences were tested using the non-parametric Kruskal-Wallis tests (p < 0.05). Significant differences are indicated with an asterisk.

The capture rates of corals exposed to microplastics were significantly lower than in the controls after 7 and 20 days (−18.00 ± 7.72 and −24.43 ± 16.42 *A*. *salina* polyp h^−1^, respectively), but not significantly different after 47 days of experiment (Table 1). The percentage of active polyps under microplastic exposure was not significantly different from the control condition after 7 days, but did differ after 20 (−2.48 ± 0.36%) and 47 days (−2.03 ± 1.37%).

For the macroplastic condition, the feeding rates were statistically lower than the control condition during the entire experiment, with very low prey capture rates (−57.10 ± 1.90 *A*. *salina* polyp h^−1^) seven days after plastic introduction (Table 1). The percentage of active polyps under macroplastic exposure was significantly lower than the percentage of active polyps in control condition after 7 days (−10.99 ± 1.98%). Activity then increased and after 20 days, polyps covered with macroplastics were significantly more active than polyps under control conditions (+7.07 ± 1.21% at day 20 and + 9.96 ± 1.89% at day 47).

Comparison between micro- and macroplastic conditions revealed that the capture rates of *A*. *salina* per polyp were significantly lower for macroplastic compared to microplastic conditions after 7 and 47 days, but not at day 20 (Table 1). The percentage of active polyps was significantly higher for macroplastics than microplastics after 20 and 47 days.

### Growth rate

**Figure 2:**
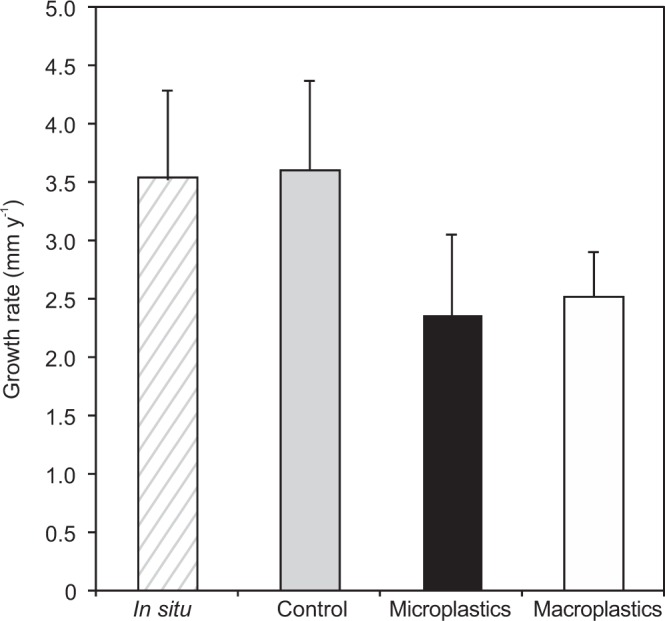
Average (±SD) skeleton growth rates (mm y^−1^) of *Lophelia pertusa* polyps *in situ* and under aquaria experimental conditions (control, microplastic and macroplastic exposure) after 69 days of incubation.

The average skeleton growth rate of corals in the control aquaria condition was not significantly different from the growth rate of corals measured *in situ* (n = 12, p = 0.763), with respectively 3.59 ± 1.90 mm y^−1^ and 3.51 ± 1.88 mm y^−1^ (Fig. 2). Coral under both macro- and microplastic conditions had a significant lower growth rates than in the control and *in situ* experimental conditions (n = 22, p < 0.001). In addition, the average growth rates of polyps exposed to microplastics (2.35 ± 1.72 mm y^−1^) were lower (n = 12, p = 0.03) than the growth rates of polyps exposed to macroplastics (2.51 ± 0.95 mm y^−1^).

## Discussion

This study shows that exposure to plastics had a significant negative effect on cold-water coral growth. The effect of macroplastics on the skeletal growth rate might be due to the physical plastic barrier that changes the hydrodynamic conditions around corals, reduces the encounter rate achieved by coral polyps and preys, which turn to reduced prey capture efficiency and therefore impairs energy acquisition^16^. Prey captures and polyp activity were lowest at the commencement of the experimental runs in treatment containing plastics, which may reflect a short-term stress response of corals, but polyp activity then increased to reach values higher than under control conditions. This increased and elevated activity may represent a behavioural compensatory response of the corals to enhance capture efficiency. Prey capture rate did increase with this increase in activity, but remained lower than in the control and the corals were not able to maintain comparable mineralization rates in the flumes containing macroplastics. Plastic obstacles seem to continually disturbed coral feeding activity throughout the entire experiment. Lower food availability has previously been shown to negatively affect physiological functions such as respiration, calcification and organic matter release in the cold water coral *Desmophyllum dianthus* in the Mediterranean Sea, with a significant impact observed after 3 weeks of starvation experiment^35^. In the present study, the reduction of zooplankton ingestion by *L*. *pertusa* polyps partially covered by macroplastic fragments decreased the dietary intake of the corals, and therefore possibly reduced the energy available for the production of three-dimensional habitat structure.

The observed increase in polyp activity during macroplastic exposure may also have been a response to lower oxygen concentrations in the micro-scale habitat formed by the interaction of plastics with corals. Long-term anoxia can alter physiological functions of *L*. *pertusa* and ultimately lead to organism mortality^36,37^. In response, *L*. *pertusa* has been shown to raise its oxygen uptake by expanding its tentacles to increase the surface of tissue in contact with seawater^37^. Finally, enhance polyp activity could also be the result of elevated mucus production triggered to clean polyps from the plastic coverage, which represents either or both physical or chemical stressors. Such a stress response behaviour has been observed previously for *L*. *pertusa* exposed to elevated sediment concentrations^38,39^. Irrespective of the cause of this increased polyp activity, the coral energy budget of exposed individuals was probably impacted by this change in feeding behaviour. Such a change has been shown in other coral species to decrease the amount of energy available for biomineralization^40^.

Microplastic exposure also reduced coral skeleton mineralization rates but, interestingly, corals behaved similarly to control colonies in terms of prey capture rate and polyp activity after 47 days of experiment (Fig. 1). As observed for shallow-water species, cold-water corals are able to ingest microplastics that have a size range comparable to their food source^41^ and incorporate them into the mesenterial tissues^14,17^. For the polychaete worm *Arenicola marina*, a chronic microplastic exposure inhibited feeding activity, which in turn reduced energy storage and the organism fitness (growth rates and survival)^16^. However, all species do not respond negatively to plastic exposure and results acquired in aquaria have shown varying results. For example, the overall energy budget of *Arenicola marina* and the mussel *Mytilus edulis* was not affected by exposure to microplastics^42^. Plastic consumption is potentially energetically expensive and could cause blockages throughout the gastrovascular cavity, or alternatively, result in a false sense of satiation in the consuming coral individual, as has been suggested in other organisms^14,43^. It is, however, unlikely that this process occurs for *L*. *pertusa* because prey capture rates after 3 weeks of microplastic exposure were similar to those observed under control conditions. However, Allen *et al*.^44^ showed recently that for the tropical coral *Astrangia poculata* exposed to microplastics, most of the plastic particles were egested in the 6 hours following consumption, but a small proportion were retained in the digestive system for at least 24 hours. Thus, the fitness of the organism could be affected by an increased energy cost caused by the egestion of plastics. Another explanation for the reduced growth rates observed in exposed corals could be that microplastics have a toxic effect on the corals. It has been demonstrated that microplastic exposure induced an inflammatory response (enhanced phagocytic activity) for the worm *Arenicola marina*^16^ and they can cause physical injuries and abrasions to shallow-water coral tissues which turn to bleaching and tissue necrosis^18^ via physical processes. Microplastics could also store and transport toxic chemicals due to their hydrophobic composition and structural complexity^45^. Thus, plastics in the digestive system of animals may impact their health via the possible release of residual monomers, catalysts and additives, and through the desorption of persistent, bioaccumulative and toxic substances absorbed from the environment^9,46,47^. Avio *et al*.^48^ have showed that microplastics coupled or not to pyrene induced genotoxic effects in *Mytilus galloprovincialis* through the production of reactive oxygen species. Finally, microplastics may additionally provide habitat niches for opportunistic pathogens^11,49^. Plastics debris could thus promote invasion of resident or foreign pathogens and enhance the susceptibility of corals to diseases such as skeletal eroding band diseases, white syndromes and black band diseases^50^. Microbiome investigations of cold-water corals are however still in their infancy^51^ and further studies are required to detail the possible transfer (and potential associated impacts) of bacteria from plastics to corals. While our study provides the first evidence that *L*. *pertusa* calcification is reduced after microplastic exposure, additional work is needed to determine the detailed processes involved. The data presented here originate from aquaria experiments with fixed environmental parameters (temperature, diet, current speed and direction), and microplastic concentrations that may be higher than the ones found *in situ*. Future studies are thus needed for an *in situ* validation of our results. It should also be noted that the replicates for each colony and condition were maintained in a same tank. This experimental design has some limitation and could explain the relatively low variation observed for some endpoints and it could influence the observed results. However, a similar set up with the same flumes was shown earlier to be useful to study *L*. *pertusa* feeding habits^52^.

This experimental work highlights hazards that both macro- and microplastics represent for coral calcification, and suggests that plastics are likely to jeopardize the resilience of the three-dimensional frameworks that cold-water coral species build. Further studies are required to fully understand the biology and ecology of the cold-water corals (e.g., reproductive cycle, larvae physiology, energy allocation and associated microbiome) and to better identify their physiological responses to plastic exposure. Considering that plastic products have been increasingly manufactured, used and discarded during the past 60 years, both local macroplastic accumulation and microplastics deriving from the fragmentation of large debris may continue to contaminate and interact with the benthic environment for centuries. Deep sea cold-water corals are generally slow growing species that may be strongly impacted by reduced biomineralization. The threat on the sustainability of their habitats could have a domino effect on the whole biodiversity supported by these engineer species.

## Material and Methods

### Coral sampling

**Figure 3:**
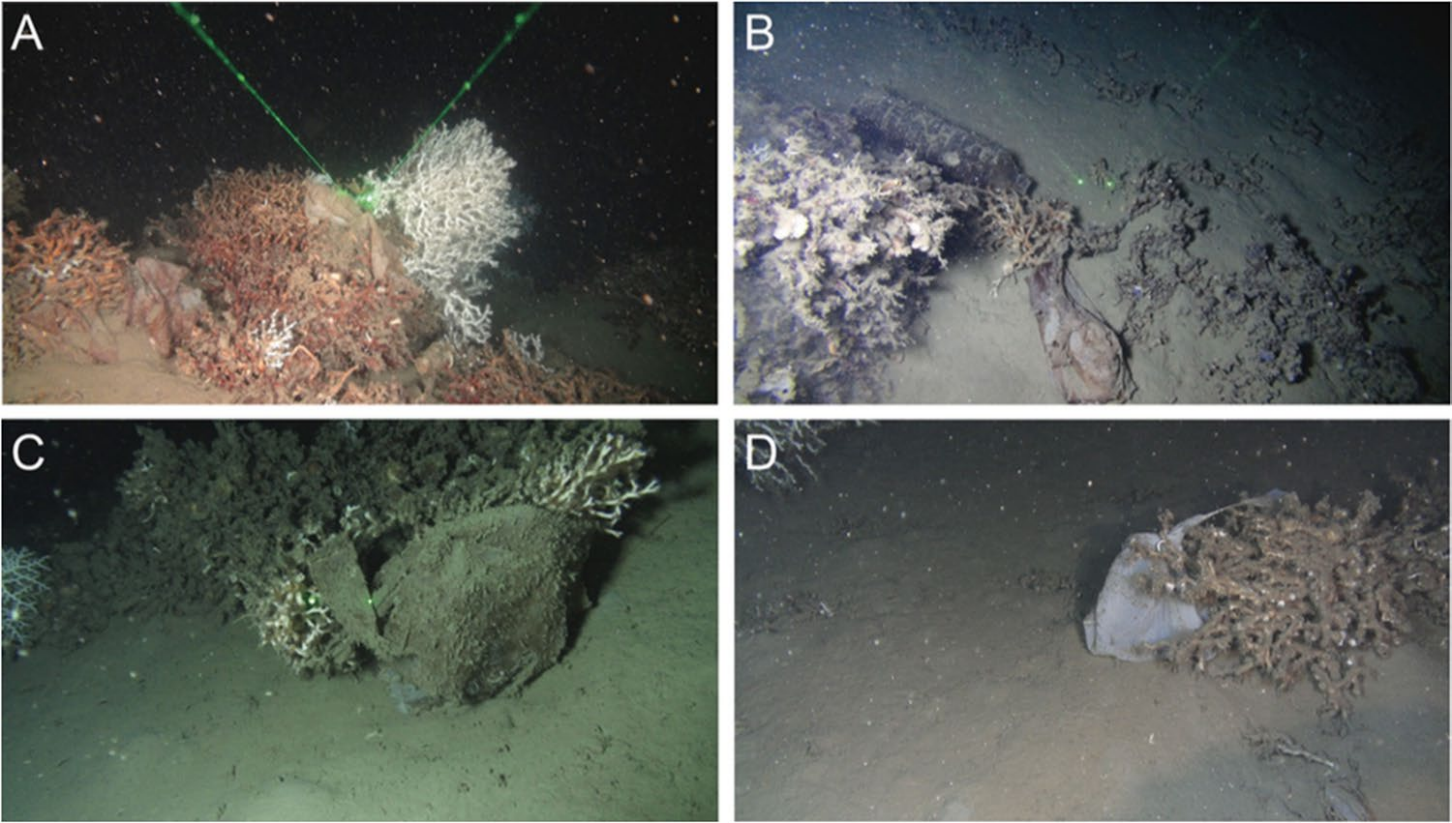
Representative views of plastic pollutions on *Lophelia pertusa* reefs in the Lacaze-Duthiers canyon, northwestern Mediterranean Sea. Different types of plastics are found in coral reefs, most of them are bags (**A**) and bottles (**B**). Dying (**C**) or dead (**D**) coral colonies covered by plastic bags. The distance between green dots is 6 cm. Fondation TOTAL/UPMC.

Sampling and imaging of corals were conducted at 540 m depth in the Lacaze-Duthiers canyon, northwestern Mediterranean Sea (42°32′72″N, 03°25′28″E) on the long-term site monitored since 2009 under the programme ‘Biodiversity, extreme marine environment and global change’ (LECOB) that had revealed large amounts of plastic debris in the canyon^53,54^ (Fig. 3). Coral branches were sampled from three distinct live colonies of *L*. *pertusa* in June 2016 using a Remotely Operated Vehicle (ROV) deployed from the R/V Janus II (COMEX company). On board, corals were transferred to aerated 30 L seawater tanks maintained at 13 °C. Once in the laboratory, corals were stabulated in thermoregulated room in the dark in a 80 L aquaria that received continuous flow (>1 renewal day^−1^) of filtered (5 μm) Mediterranean seawater pumped from 5 m depth^55^.

### Plastics

Polyethylene is the most common plastic type used in the world and is found in oceans as micro- and macro-debris^1,56,57^. The macroplastics used in the experiment were 10 cm × 10 cm sized pieces of low density polyethylene (LDPE) meant to represent pieces of plastic bags. The macroplastic sizes were chosen to cover the entire height of the coral branch. The microplastics were composed of 500 μm LDPE microbeads (Santa Cruz Biotechnology Inc., Dallas, USA). The microplastic size corresponds to the size of the zooplankton commonly used to feed corals in aquaria^58–61^. Before the start of the experiment, both macro- and microplastics were incubated for two months in 5 L seawater tanks continuously supplied with filtered Mediterranean seawater. The goal of the pre-incubation was to allow colonization by environmental bacteria, a phenomenon observed in natural environments.

### Experimental design

Experiments were conducted in recirculating flumes of 58 L each designed to maintain a regular and user defined rate of water circulation^52^. The flumes were maintained in the dark at 13 °C ± 0.5 °C in a thermoregulated room. A motor (Modelcraft) driven propeller maintained a constant flow of 2.5 cm s^−1^ in each flume. Each system was semi-closed with a continuous supply of 2.5 L h^−1^ of oxygenated, thermoregulated and filtered (5 μm) Mediterranean seawater (corresponding to one renewal per day). A 180 μm mesh at the spillway retained particles within the tank. Three different flumes were used to maintain three different experimental conditions for 2 months: one control, one with microplastics and one with macroplastics (Supplementary Fig. S1).

A total of 15 coral nubbins from the three distinct sampled colonies (each nubbin containing 4 to 33 living polyps) were randomly assigned to use within each flume. Nubbins were fixed to cement blocks using an aquatic epoxy resin^62^. Corals were acclimated in the flumes for four weeks before the addition of plastics. For microplastic conditions, the beads were added to the flume at a concentration of 350 beads L^−1^, which corresponds to the concentration of the zooplankton preys commonly used for regular feeding within the laboratory. The microplastic to zooplankton ratio that we use has been seen in the Mediterranean Sea surface waters with microplastic concentrations ranging from 0 to 2.28 mg L^−1^, and polyethylene being the most common plastic^63,64^. However, there are no precise quantifications of microplastics in the deep-sea in general, and in the Lacaze-Duthiers canyon in particular. Considering that not all the surface microplastics reach the sea floor, our experimental concentrations may be higher than *in situ* concentrations. Our results must thus be considered in the light of that caveat. For macroplastic conditions, the plastic pieces were placed in the flumes to partially cover (~50%) living polyps over the entire height of the coral branch, simulating conditions that we observed in the field (Supplementary Fig. S1) and that have been reported by others on deep sea corals in the Mediterranean Sea^28,53,54^. The macroplastics covered the living polyps that faced the current but not the polyps that were on the other side of the coral branch. Corals were fed three times a week alternately with freshly hatched *Artemia salina* nauplii (350 *A*. *salina* L^−1^) and with Marine Snow plankton diet (Two Little Fishies Inc, Miami Gardens, USA, 5 mL per flume), to provide a complete and diverse nutrient supply^36,61^.

### Prey capture rate

Coral capture rate was measured 7, 20 and 47 days after the start of the experiment using a method published by Purser *et al*.^52^. Triplicate water samples (100 mL) were taken each hour after feeding over a 4-hour period and filtered on 55 μm mesh. *Artemia salina* nauplii were counted on the mesh with a binocular magnifier to calculate the concentration of suspended *A*. *salina* remaining in each flume. The number of zooplankton prey removed by coral capture was normalized against the number of living polyps present in the flume. Control measurements were also conducted in flumes containing no corals to quantify zooplankton sedimentation. The consumption of *A*. *salina* per polyp was corrected against control measurements. Most of the zooplankton consumption by *L*. *pertusa* occur during the first hour following prey delivery^52^ (Supplementary Fig. S2), and our results thus focus on this time period.

### Polyp activity

Cold-water coral polyp behaviour can be studied by video monitoring^65,66^. We applied this approach to measure polyp activity using two-hour video captures. Two cameras (GoPro, San Mateo, USA) were installed in each flume. To maximize the total number of polyps observed, one camera filmed a lateral view and the other an aerial view of the corals. The activity rates were measured by analysing differences between consecutive images extracted from the videos^67^. Three subsamples of 20 images were extracted every 6 minutes from the video and combined into an image stack. Optimisation tests showed that the chosen time interval and number of images allowed to determine the highest number of active polyps for the duration of the sequence (Supplementary Fig. S3). The percentage of active polyps was calculated by counting the total number of moving polyps in each view using the Image J v1.51 software and by dividing this number by the total number of polyps.

### Coral growth rates

Sclerochronological analysis was used to measure coral growth rates, after 2 months of exposure, on three replicate nubbins from each colony (each containing 5 or 6 living polyps) for each experimental condition. Polyps were labelled with a fluorescent calcein staining (150 mg L^−1^) at the start of the experiment as described in Lartaud *et al*.^68^. At the end of the experiment, the coral nubbins were cleaned for 12 hours in a hydrogen peroxide solution (H_2_O_2_, 4%) at 60 °C to remove organic tissues, and then rinsed in demineralized water. Each polyp calyx was then separated from the others, embedded into epoxy resin Sody33 for 24 hours and cut into slices with a Buehler Isomet low-speed saw. The coral sections were mounted on slides with Araldite© resin, abraded and then polished, for subsequent observation under an epifluorescence microscope (Olympus IX51) with an excitation at 495 nm. The new skeleton formed between the calcein marking (i.e., beginning of the experiment) and the summit of the septum of the calix (i.e., date of death) was measured using the ImageJ© software (repeated 5 times) by superposing the fluorescence pictures, which revealed the stain, and the optical light pictures showing the coral skeleton morphology^68^ (Supplementary Fig. S4).

To compare experimental and *in situ* growth rates, an analysis was also conducted on *L*. *pertusa* fragments that had been marked and redeployed in their natural habitat for ca. 2 months. The *in situ* corals used originated from an earlier study^69^ conducted at the same location in the Lacaze-Duthiers canyon and for the same duration.

### Statistical analysis

Tests for normality of variance were performed using the Shapiro-Wilk test on R software (v3.4.3), which revealed that the distribution was not normal for feeding rates, percentages of active polyps or skeleton growth rates (p < 0.05). A multiple-comparison non-parametric Kruskal-Wallis test was thus used to analyse the differences between the three experimental conditions and for differences between 7, 20 and 47 days for each parameter investigated.

## Electronic supplementary material

Supplementary figures
