# Supplementary material for: Macro- and microplastics affect cold-water corals growth, feeding and behaviour
Source: Sci Rep. 2018 Oct 17;8:15299. doi: 10.1038/s41598-018-33683-6 (PMC6192985; doi:10.1038/s41598-018-33683-6)
Supplement: Supplementary file 1 — Supplementary figures [file 41598_2018_33683_MOESM1_ESM.pdf]

Macro- and microplastics affect cold-water corals growth, feeding and behaviour

Chapron L.[1], Peru E.[1], Engler A.[1], Ghiglione J.F.[2], Meistertzheim A.L.[2], Pruski A. M.[1], Purser A.[3], Vétion G.[1], Galand P. E.[1], Lartaud F.[1*]

Supplementary figures

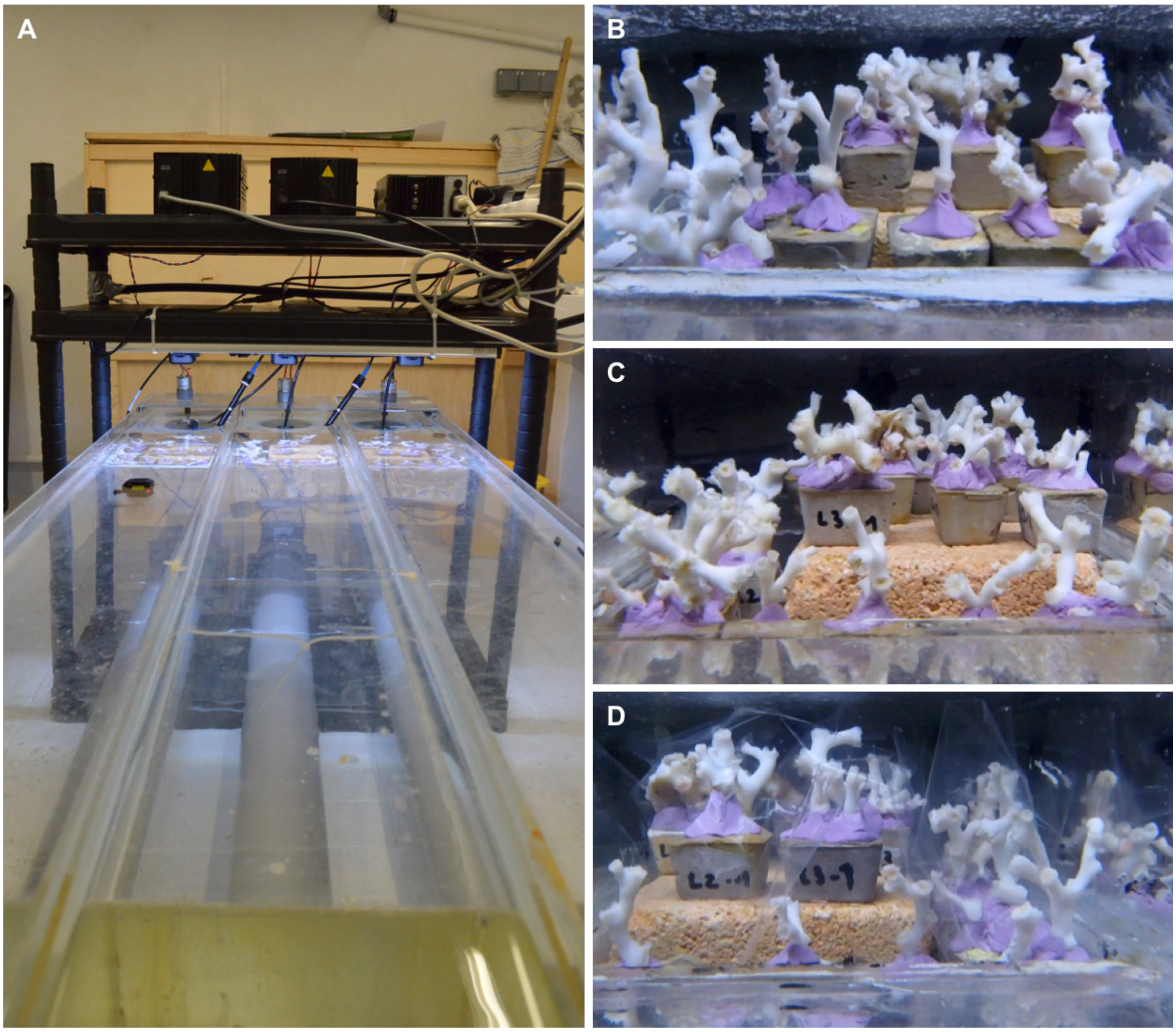

Figure S1: Recirculated flumes used for the experiment (A) and *L. pertusa* nubbins under different experimental conditions: (B) microplastics, (C) control and (D) macroplastic.

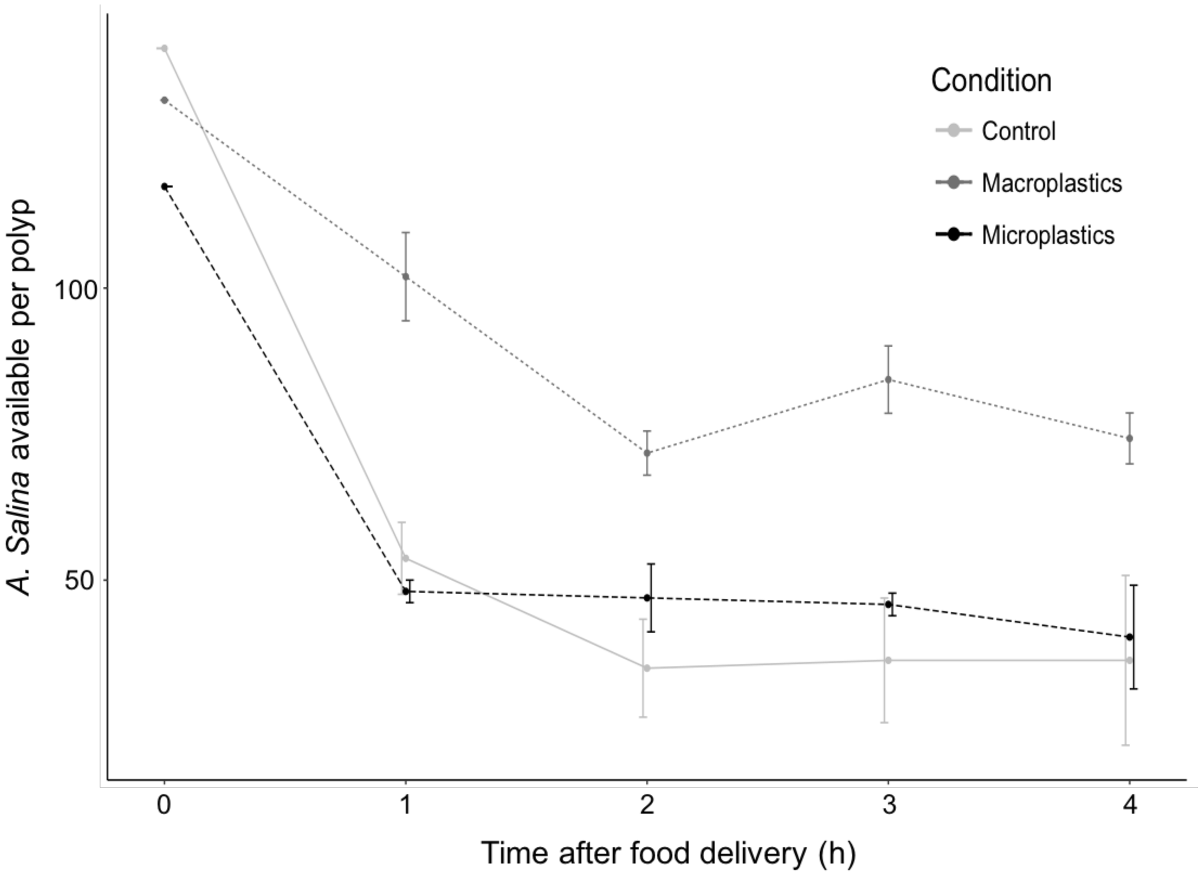

Figure S2: *A. salina* available per polyp for *L. pertusa* during four hours following feeding under each experimental condition (control, microplastics, macroplastics).

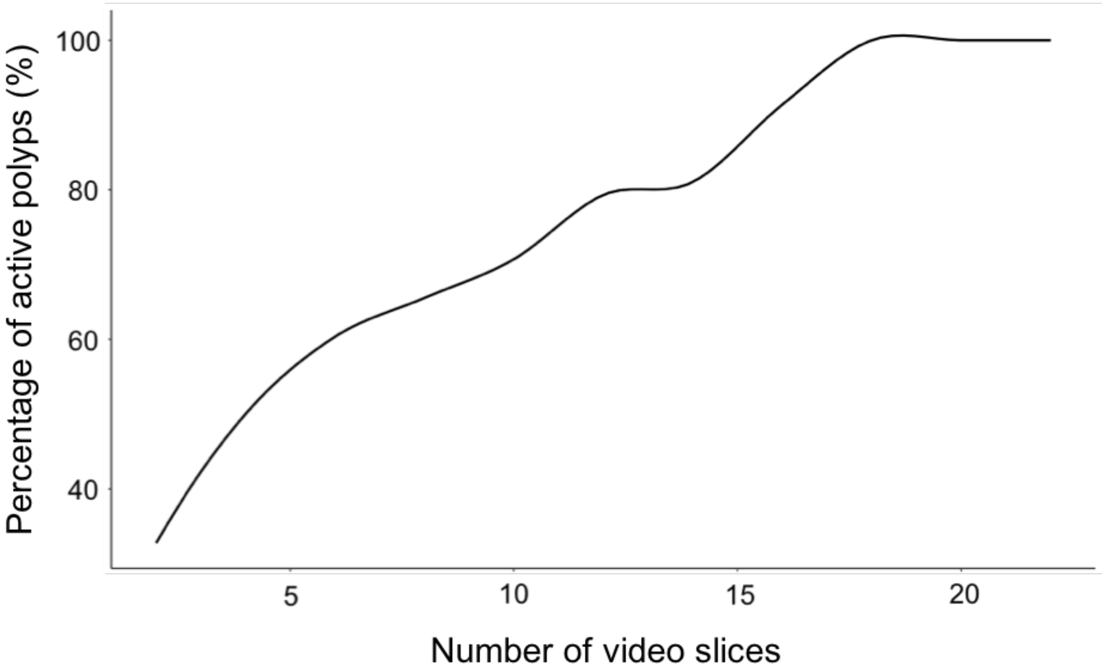

Figure S3: Percentage of actives *L. pertusa* polyps in relation to the number of video slices selected within the

video filmed for 2 hours after feeding.

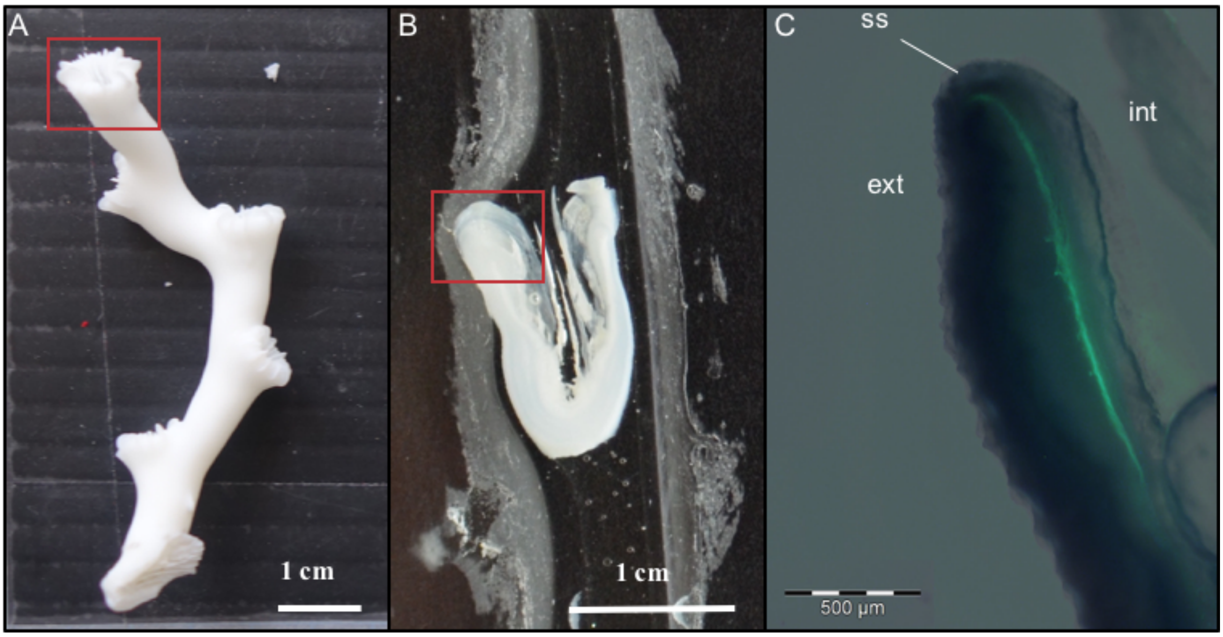

Figure S4: (A) Fragment of *L. pertusa* containing five polyps with (B) the highest one (red scare) cut into a section mounted on slide of which (C) a major septum (red scare) is observed under fluorescence revealing the calcein staining (green line). ext = extern, int = inside the polyp, ss = summit of the septum.
